# Invisible Labor and the “Ghost Particle”: Underground Physics at the Kolar Gold Fields**

**DOI:** 10.1002/bewi.202400020

**Published:** 2025-04-16

**Authors:** Nithyanand Rao

**Affiliations:** ^1^ Department of Communication and the Science Studies Program University of California San Diego USA

**Keywords:** invisible labor, verticality, colonialism, race, caste, laboratory studies

## Abstract

When cosmic rays—high‐energy particles from outer space—encounter the Earth's atmosphere, they produce particles called neutrinos. To detect them, physicists go underground inside deep mines where the overlying rock can filter out the cosmic‐ray background radiation. I examine how the first such detection of neutrinos in 1965 by an international team of physicists at the Kolar Gold Fields (KGF) in India—a gold mine where the British began mining in 1880—was made possible by the invisible labor of lowered‐caste, or Dalit, miners. By studying the underground, this paper contributes to recent attention to verticality in the history of science, while moving away from the dominant approach to spatial studies of sites of science, the lab–field framework, and instead examining the social, political, and economic conditions that made KGF, with its depth, possible as a site for physics.

Using labor histories of KGF and archival material about the experiments, I argue that the mines became nearly three kilometers deep only because of a regime of racialized labor in which Dalit miners worked in difficult and dangerous conditions for less than subsistence‐level wages. I also show how the experiments depended on this invisible labor that ran the mines.

## 1 Introduction

In August 1965, Mambillikalathil Govind Kumar Menon invited his mentor, the British physicist Cecil Frank Powell, to visit the Kolar Gold Fields (KGF), a gold mine in south India, the next month.^1^ Menon and colleagues from the Tata Institute of Fundamental Research (TIFR) in Bombay (now Mumbai), India, were working in an underground laboratory at KGF with physicists from the United Kingdom and Japan, and they had just detected elusive particles called neutrinos that are produced when cosmic rays—high‐energy particles from outer space—interact with the Earth's atmosphere. The KGF team shared the credit for this discovery with a competing experiment at the East Rand Proprietary Mines (ERPM) in South Africa, led by the U.S. physicist Frederick Reines, that had also made the detection earlier that year. The two teams of physicists had chosen KGF and ERPM, the two deepest mines in the world at the time, because finding the “atmospheric” neutrinos required a space that was free of the background radiation of the cosmic rays that produced them. At KGF, the rock overlying the laboratory, 2.3 kilometers underground at the 80th level of the Heathcote shaft of the Champion Reef mine, filtered out cosmic rays and allowed only the neutrinos, which rarely interacted with matter, to pass through. And Menon wanted Powell to visit this lab. Powell was keenly interested in the work of the physicists at TIFR, and Menon often sought his mentor's advice.

To arrange for Powell's visit, Menon wrote to the director of the KGF mines. In this letter, Menon mentioned something else: “I realize that on account of the strike situation you have a number of serious problems on your hands.”^2^ The miners at KGF were striking work at the time. Just two days later, Menon informed Powell by telegram that the labor strike meant that his proposed visit had to be canceled.^3^ Without the miners, the physicists couldn't go down the mine and, without the mines functioning, the groundwater that flowed into the tunnels couldn't be pumped out. The miners had been on strike earlier that year as well, in May, for the same reason: they were, according to a news report, “protesting against the supply of inferior quality rice by the management and also against the increased price of rice.”^4^ The year 1965 was one of drought, causing a severe shortage of food grains and urgent imports from the United States.

Later that year, the physicists encountered other difficulties as they continued their experiments. The lead British collaborator, Arnold Wolfendale of Durham University, wrote to Menon in November 1965, mentioning a fire at KGF which he was “most disturbed to learn about.”^5^ Wolfendale did not say if there were any casualties. A month later, he wrote that the “delay now that we have telescopes ready for installation is maddening.”^6^ Menon replied that he had “also been disturbed about the fire at KGF and the time scale involved in getting things to normal.”^7^ The Indian government, he added, had other priorities such as the famine and a war with Pakistan.

The correspondence above contains elements not typically part of histories of physics: gold mining, labor strikes, accidents, the larger political economy. Are these elements incidental to the neutrino experiment at KGF? I show that they are not, and that they can tell us much about the social, political, and economic conditions that made possible not just the experiments at KGF but also those at other deep mines around the world that have been—and continue to be—important for contemporary physics.

## 2 Beyond the Lab and the Field

For historians of science, a spatial approach to studying sites of science has meant a focus on spaces known as laboratories or field sites. A lab, by virtue of its standardization that makes it commensurable with other labs, facilitates erasing the local specificities of the site that make producing science possible, thereby helping scientists claim the results of their work as “placeless.”^8^ By contrast, a field site is one where the place, far from being erased, is crucial to the value of the knowledge produced.^9^

In this scheme, physics is usually seen as a laboratory science. But then why have historians of physics not studied underground laboratories? I argue in this paper that the very notion of the laboratory as deployed in the scholarship presents a blinkered view of what a space for science is, making an underground lab seem unexceptional. But a lab underground, as the physicists’ correspondence above shows, is not the same as one above ground. If, like at KGF, it is inside a mine, we must ask why a mine came to be in that specific place, how physicists came to see it as suitable, and what kept it running.

Adriana Minor's study of cosmic ray physics in Latin America,^10^ in a shift from the spatial approach that historians have favored, such as studying laboratory architecture,^11^ highlights how there is a diversity of sites that are relevant for physics experiments, such as high‐altitude field stations and hot air balloons.^12^ Her study emphasizes the role of “verticality” in the study of cosmic rays, focusing on altitude. But verticality is also about depth. How might we study underground labs, and what might they add to our understanding of contemporary physics?

To begin with, there is a problem with Minor's premise of studying “non‐laboratory spaces and practices,”^13^ which is that it rests on the historian judging what is a lab and what is not—using it as an analytic category—or on analyzing the implications of scientists’ labeling of a space as a lab or a field site. Historians too often fail to do the latter—interrogate the lab or the field site as actors’ categories—and instead uncritically use them as analytic categories. There is often a predilection to slot spaces into these pre‐defined categories, through representing the field site and the lab as “open and enclosed sites of knowledge production,”^14^ respectively. Such assumptions reveal how scholars conceptualize the field site as everything the lab is not—unbounded, outdoors, uncontrolled—creating a “laboratory–field dyad,”^15^ a binary of ideal types.^16^ But such a view is unhelpful,^17^ not least because these categories become “scripts” that dictate what we pay attention to and how we study sites of science.^18^

For instance, the lab–field framework has implications for how we link, or fail to link, sites of science to coloniality. Field research in nineteenth‐century astronomy to observe solar eclipses, for example, depended on colonial networks for expeditions to islands in the Caribbean and the Pacific.^19^ And field sciences such as ecology remained, even in the mid‐twentieth century, “deeply implicated in projects that were colonial under other names and in novel forms.”^20^ Despite this evident coloniality, Megan Raby^21^ notes how historians of science miss noticing crucial elements that can tell us what enables scientists to choose certain places for their work—such as a field site's location on colonized land.^22^ Further, having displaced on to the field site any conceptual links that knowledge production has with place, we miss how labs, too, may be situated on colonized land.^23^ In this way, questions such as why a lab or a field site is located where it is, and what political, economic, and social conditions made the site available to scientists rarely arise in the lab–field framework when they are used as analytic categories.

But to uncritically use these categories as historical actors do, to think of a space as only a lab or a field site, can also present the same problem—it obscures what allows a space to be used by scientists. These categories predispose us to center scientists’ practices to the neglect of other practices that may in fact have historically constituted—and continue to constitute—the space. For example, as Minor points out, historians have not studied cosmic ray research with a focus on spatial questions, on its own terms.^24^ Instead, it has been associated with the “charm of natural explorations.”^25^ Physicists’ narratives may present such sites as exotic, but there is no reason for historians to not dig deeper. Such oversight becomes particularly consequential when we study spaces that were built, and were kept functioning, for other purposes that scientists only later represented as a lab, as is the case with physics experiments performed in underground mines. To transcend these limitations of the lab–field framework, I keep these categories aside and instead ask what conditions made certain sites available to physicists. To tell the story of the site of a physics experiment with only what physicists did is to effectively treat the site as natural, pre‐existing, and materially ahistorical. When it comes to mines such as ERPM and KGF, we must instead examine the historical reasons why they are located where they are and what made them suitable for physicists. Why were the deepest mines in the world—and thus the physics experiments that discovered the atmospheric neutrino—located in South Africa and India? In this paper, I focus on KGF.

## 3 The Neutrino Experiment at KGF

Physicists from TIFR first arrived at KGF in 1950,^26^ and studied how the cosmic ray flux attenuated with depth underground.^27^ They returned in April 1961,^28^ later joined by Japanese collaborators, and over the next two years, as they went deeper, they observed that placing detectors nearly three kilometers underground allowed the overlying rock to completely filter out muons, particles that the cosmic rays produce on interacting with the Earth's atmosphere.^29^ They realized that such a space could be used to study neutrinos that are also produced in the cosmic ray shower. The little‐studied particle had been proposed by Wolfgang Pauli in 1930 to preserve the laws of conservation of energy and momentum which were seemingly being violated during the radioactive decay of unstable elements.^30^ The neutrino was first detected by U.S. physicists Frederick Reines and Clyde Cowan in 1956, being emitted from a nuclear reactor.^31^ But the ones that were, according to theory, produced by cosmic rays hadn't been detected.^32^

Thought to be massless at the time, the neutrino interacts only through the weak nuclear force, which allowed it to pass through matter, including rock—hence its moniker, the “ghost particle”—and reach underground chambers at KGF where the cosmic rays could not. But the rock was more than a filter; it was also part of the detector. On the rare occasions when the neutrinos interacted with the rock, they produced muons, and *these* were the particles that the physicists hoped to detect. This requirement—distinguishing muons produced by neutrinos interacting with the rock from the muons produced directly by cosmic rays—made KGF, with its depth, suitable.

After reading early work from the TIFR group,^33^ Reines, too, realized that such an experiment could be done at KGF. He travelled there to set up an experiment, initially with no intention of collaborating with TIFR and, when pressed, willing to include them but only with himself leading the collaboration. This did not please Homi Jehangir Bhabha, the founder of TIFR and India's “science czar.”^34^ Bhabha refused permission,^35^ and Reines went to ERPM instead to collaborate with South African physicist Jacques Pierre Friedrich (“Friedel”) Sellschop. The two teams of physicists both detected atmospheric neutrinos in 1965.^36^

The reason these physicists sought deep mines is evident in a letter Reines later wrote, in 1968, during an inquiry by the Atomic Energy Commission (AEC) into his work in apartheid‐era South Africa. Reines had employed Black African men who were the miners at ERPM to excavate a chamber more than three kilometers underground, and the AEC wanted details of the conditions under which they had worked. He also faced protests at his university for having collaborated with the South African government. In his response^37^ to an open letter that other faculty in the university had written to him, Reines explained just why a deep mine was necessary:

The experiment is being conducted there because there exists no other such location in the entire world in which to carry on these scientific cosmic ray investigations. Were we to prepare a comparable location in the U.S. it would cost the resources of the magnitude required to build a small university.

Reines was referring to how ERPM's depth made unnecessary “elaborate anticoincidence arrangements” to distinguish the neutrino signal from cosmic ray noise.^38^ A deep mine effectively subsidized the experiment. For my purposes in this paper, I do not examine this claim or how Reines, who states in this letter that ERPM was the only possible location for the experiment, had earlier claimed the same status for KGF, nor do I examine the details of the two experiments. I am concerned merely with the fact that the two teams of physicists chose KGF and ERPM for their experiments, primarily because these mines were deep. What made them so deep and what kept them functioning, making the physics experiments possible? In the next section, I address this question for KGF.

## 4 Deep Mining at KGF

The British began large‐scale mining at KGF in 1880 in what was then the princely state of Mysore (now Karnataka) in south India. Investors set up many joint stock companies in London, of which five survived the turn of the century, including Champion Reef, and their mining operations were run by the London‐based firm John Taylor & Sons. But they needed more laborers willing to do dangerous work for low wages than were available in the area. The mining companies therefore sought laborers from neighboring districts of the provinces of Madras and Travancore (now the states of Tamil Nadu, Andhra Pradesh, and Kerala). Most of them were from lowered castes, and this was not accidental.^39^ ^,^ ^40^ The men who arrived were landless agricultural laborers, fleeing drought, famine, and unemployment. They were also fleeing caste oppression, since they were *Adi Dravidars*, “untouchable” castes who are today known as Dalits. They not only faced discrimination but were effectively slaves to dominant‐caste landholders in their native villages.^41^ At KGF, by contrast, they were able to fashion for themselves new identities in relative freedom.^42^ KGF was, for instance, among the places where the Tamil anti‐caste intellectual Iyothee Thass worked in the late nineteenth and early twentieth centuries.^43^ And caste was central to the working of KGF.

### 4.1 The Political Economy of Gold Mining at KGF

The physicists’ letters that we saw in the Introduction provided but a glimpse of the dangerous working conditions in the deep mines. The rock temperature three kilometers underground exceeded 65 °C,^44^ and the dust was hazardous. Accidents and fires were an everyday occurrence, as were “rockbursts,” collapses of underground tunnels so violent that they were felt above ground. There was an average of two hundred accidents a year, often fatal ones, and around 3,400 workers lost their lives underground over a century of mining, at a rate worse than South African gold mines.^45^

What made mining possible and profitable in these conditions? The British were prospecting for gold throughout their empire, and mining at KGF was part of the “gold rush” in various parts of the colonized world. These gold rushes supported, and were spurred on by, the adoption of the international monetary gold standard in the nineteenth and early twentieth centuries. The gold standard, officially adopted in Britain in 1816,^46^ meant that the value of its currency unit was fixed to be equivalent to that of a specific weight of gold; or, to put it differently, the price of one unit of gold was kept fixed.^47^ Given the extent of Britain's empire and influence, more countries joined the gold standard beginning in the 1870s; the U.S. joined in 1900. These were the two countries with the largest gold reserves.^48^ Their expanding economies, particularly before World War I, required more money to keep entering circulation. And since the currencies were tied to gold, the demand for the metal was insatiable. Despite this demand, the price of gold could be held constant because the U.S. and Britain added vast quantities of it to their reserves by mining in lands they had colonized: Native American land in California and other states in the U.S., beginning in mid‐nineteenth century, and for Britain its colonies Australia, South Africa, and India.^49^ Gold price remained constant until World War I, and then remained more or less constant again until 1931 when Britain formally left the gold standard.^50^ The U.S. followed in 1933, but the gold price continued to be held fixed.^51^ After World War II, the Bretton Woods agreement tied exchange rates of other currencies to the U.S. dollar, which in turn was tied to gold, thus “reaffirming gold's status as the foundation of global systems of value.”^52^ This linkage continued until 1971, when the price of gold, no longer tied to currencies, began rising dramatically.

Most of the gold produced in India in this period was from KGF. By 1915, the intense mining had exhausted the high‐grade ore available at moderate depths,^53^ giving way to low‐grade ore which had to be mined at greater depths, and the mining shafts eventually reached more than three kilometers deep.^54^ Mining at such depths, and the fact that more low‐grade ore had to be extracted to produce a given amount of gold, entailed a higher production cost. This cost couldn't be recovered from selling the gold because its price was held constant by the international monetary gold standard.^55^ Given that the mining companies’ wage bill was always greater than 50 percent, sometimes up to 75 percent,^56^ of their working expenses, it was the availability of cheap labor that kept the mines running as the shafts went deeper.

We can understand the nature of the gold mining industry's relationship to labor from the point of view of the employers from the *Report of the Minimum Wage Committee in 1950* appointed by the Mysore state.^57^ In a section titled “Capacity of the Industry to Pay,” the report quoted from the *South African Mining and Engineering Journal*:

Gold bearing ore is a national asset only so long as the cost of producing the gold is less than the value of the gold produced, that is to say, so long as the ore is payable. The dividing line between payable and unpayable ore is determined by the working costs of the mine concerned.^58^

This dividing line, the depth at which the cost of production equaled the value of the gold produced, is called the pay‐limit. If the pay‐limit rose—meaning a high cost of production which allows for mining only at shallow depths—it made gold below that level unattainable. This, according to the *Journal*, “deprives the country of part of its national assets, reduces its capacity for employment and has the same effect as living on capital.”^59^ Further:

Every reduction of the pay‐limit, even by as little as a tenth of a penny‐weight, increases materially the wealth‐producing capacity of the country's gold endowment. Such marginal ore, once passed, may be difficult or impossible to work afterwards.^60^

The Mysore state's committee admitted that there is the “other point of view,” which is that “reduced wages should not be resorted to” for making more ore payable. This is because “an industry which is unable to pay a minimum wage has no right to exist.”^61^ But such an industry did exist, and it was not until the workers at KGF were able to collectively bargain that their wages increased.

### 4.2 Caste and the “Racial Pyramid”

But there was more to suppression of wages at KGF than just the pay limit. Even as the mines went deeper in the 1930s, and the price of gold—and thus profits and shareholder dividends—increased as Britain abandoned the gold standard in 1931,^62^ the miners’ wages were still what they were before World War I. In fact, the inflation in the inter‐war years meant that their wages had *decreased* in real terms—as it had when the mines were at the peak of their profitability in the years 1902–1912 and 1934–1941.^63^ What factors other than the gold price suppressed the wages?

The Mysore state, with its upper‐caste rulers, was crucially dependent on royalty from the mining companies and permitted them to effectively run a state within a state, colluding with them in enabling the exploitation of the miners.^64^ For example, trade unions were legalized in Mysore only in 1942, whereas the provinces of directly British‐ruled India had done so in 1926. And it was the trade unions, after many strikes and protests, that forced the state to determine minimum wages by forming its committee in 1950.

There was also racial disparity in wages. Before strikes forced an increase in wages in the 1940s, the average monthly wage for an Indian employee was less than £3,^65^ with the lowest wages being for “unskilled” laborers who were paid less than half this figure.^66^ Meanwhile, the wages for European employees did increase consistently, even when the gold price was low, and more so when it increased in 1931. The average monthly wage for European employees in the 1930s was nearly £50.^67^ The racial disparity in average monthly wages was thus nearly 16 : 1 but had once been as high as 22 : 1 (in 1917). For comparison, the racial disparity in average wages in South African gold mines was never higher than 12 : 1.^68^ In terms of real wages, the Indian miners’ wages in the 1940s had decreased by about a third from 1900.

The principal factor in this suppression of wages was caste. After labor shortages in the initial years, there were more people than jobs at KGF, particularly after 1900. Initially the five competing mining companies offered good wages, but once John Taylor & Sons took over the management of all the mines and the labor supply became abundant, the labor market became a monopoly, and wages could be kept fixed. As mining intensified, KGF became the largest employer in the state of Mysore: it had close to 35,000 miners in 1907, during the period when production was at its peak.^69^ There were few other jobs available in the area and, for the newly arrived, working in the mines was preferable to going back to the caste oppression of their native villages. For these reasons, the mining companies effectively had a captive labor pool. They facilitated this creation of a standing labor force by building mining towns that provided housing and basic amenities for a significant fraction of their permanent employees. This was important if the mines were to run around the clock, with the miners working in three eight‐hour shifts to maximize profits. Living in “coolie lines”^70^ in the mining towns also meant that later generations of KGF's residents took up mining, some after training in the mines’ workshops, thus reinforcing and reproducing the caste‐bound nature of the mining work. KGF's townships were also a “racial pyramid.”^71^ The residential areas, schools, and other facilities for the Dalit miners were segregated from those of the mining company's managers and engineers—British and later “neo‐colonial Indians,”^72^ or in other words, upper‐castes—who lived in bungalows leading a life of genteel luxury, leading some to call KGF “Little England.”^73^ Notably, the TIFR physicists, too, were all upper caste.

Until the 1940s, up to two‐thirds of the labor force was made up of contract laborers rather than permanent employees. This proportion declined over time to about half, until contract labor was done away with in 1949.^74^ The contract laborers did not have any benefits other than their wages. They did the most dangerous jobs in the deepest levels of the mine and often worked double shifts to earn more, leading to exhaustion and accidents, even death. This did not trouble the mining companies since they always had a plentiful supply of men who arrived seeking jobs.

We can thus see what enabled KGF to acquire great depths. The hierarchies of caste and race, combined with the constraint imposed by the fixed gold price, made possible the extraction of gold with low‐wage labor despite the dangerous working conditions. And the depth thus acquired is what made KGF conducive for the later physics experiments. Therefore, if KGF's depth was necessary for the physicists’ detection of the neutrino, so were the social, economic, and political conditions that made mining at depth possible. Further, as we saw from the physicists’ correspondence, the regime of labor at KGF did more than just prepare the ground for the experiments—it was necessary for their running too.

But the miners’ labor was not just digging. Mining at KGF needed the skills and knowledge embodied in the miners’ practices. The official sources label the miners as “unskilled,” but the historian Janaki Nair^75^ notes how the songs they used to sing while at work show the knowledge embodied in their practices. She alludes to what is called “pitsense” in mining parlance—the ability of miners to sense, and to be attuned to, their environment as they go about their work underground. For example, they needed to acclimatize to working at depth in the heat and dust and stay alert for signs of impending rockbursts. They navigated the underground spaces, sometimes without light, for they tried to save for themselves the meagre supply of candles, and later carbide lamps, that the mining companies provided. Any reference to the KGF miners’ skills, however, is absent from the official record but for passing mentions that “the historian has to strain her ear” for.^76^ One example is how the *Bulletin of the KGF Mining and Metallurgical Society* carried an article that remarked upon the “extraordinarily small number of blasting accidents in proportion to the number of holes drilled.”^77^ Such indirect acknowledgement of the miners’ skills went against the mining companies’ interest in blaming the miners for accidents and for their inefficiency, and was, notes Nair, suppressed thereafter from the *Bulletin*.

## 5 Conclusion

KGF was host to other experiments after the discovery of the atmospheric neutrino, prominently one that attempted to detect the theoretically predicted decay of protons beginning in 1980. However, in 1992, the mines—and the physics experiments they housed—shut down. As mining was no longer viable, the public sector mining company could not pay for the electricity to pump out water and prevent the mines flooding, and neither could TIFR. KGF closed formally in 2001, after more than a century of mining.

I have argued in this paper that a spatial approach that takes too seriously the lab–field framework constrains our inquiry, and that this is especially evident when we consider underground mines used for physics experiments. We must instead ask what made such spaces available to physicists. Doing so brings into view elements usually missing from histories of physics: colonialism, race, caste, labor, and the larger political economy.

Such conjunctures are not confined to KGF. One could do a similar study of ERPM in South Africa, with its Black miners, many of them migrants from elsewhere in Africa.^78^ Then there is the Homestake gold mine in South Dakota, the largest and deepest mine in the U.S., which appears in Trevor Pinch's book, *Confronting Nature*.^79^ The book is a sociological study of the controversy over the results of the solar neutrino experiment at Homestake beginning in 1965. Pinch notes that the site initially considered for the experiment was a silver mine in Idaho, but it was eventually not chosen because of “a contractual problem”: the contract from the U.S. Atomic Energy Commission to the mining company had a “general non‐discrimination clause.” Pinch quotes Raymond Davis, the physicist leading the experiment, who explains: “Idaho apparently has had a reputation […] for not being too kind to black people.”^80^ If the team performing the experiment had Black people, they would have had trouble visiting the mine.

When the Homestake mine was eventually chosen, there was another problem. Pinch quotes from a letter to the physicists written by an AEC official that stated that there was a “difficulty which might affect the long‐term prospects for the experiment”:

It should be noted that the workers at Homestake have just become unionised for the first time in their history. The long‐term effect of this on the experiment cannot be anticipated at this time. I am told that the mine is not a highly profitable operation since the ore now being used runs $10 of gold/ton. Higher labour costs could possibly cause the mine operation to be shut down before all the experiments are completed. ^81^

If we dig deeper into the conditions that made the experiment possible and attend to the lab's history, we find that it is located on land violently stolen by white settlers from the Lakota Sioux and Cheyenne tribes in the Great Sioux War of 1876.^82^

We could dig into the histories of other mines that have hosted, or continue to host, underground physics experiments. The Creighton mine in Sudbury, Canada, where mining for nickel and copper by immigrant laborers began in the 1880s, was a site of extreme pollution with heavy metals and sulfur dioxide. Kamioka in Japan, the site of many important neutrino experiments, was also a prisoner‐of‐war camp for Allied soldiers during World War II, who were used effectively as slaves to mine lead, zinc, and gold, among other metals.^83^ These are metals that were needed during Japan's imperial expansions and conquests in Asia, and the cadmium in the mining waste caused one of the worst mass poisonings in Japanese history in the early twentieth century. The physics experiments conducted in all these mines mentioned above collectively led to the discovery of the phenomenon called neutrino oscillation, implying that the particle has mass, contrary to theoretical expectation. This ranks among the most consequential—and unexpected—experimental results in contemporary particle physics, posing a challenge to its theoretical framework, the standard model, which is still unresolved. Among the efforts to study this question is a new experiment that is being built inside Homestake mine.^84^

It is not accidental that these different mines, and the experiments they later hosted, are located where they are—they are products of colonial and imperial violence. Looking beyond pre‐conceived categories and paying attention to depth, therefore, opens a hidden door that reveals the spatial and material conditions of possibility for contemporary physics.

## Footnotes

n1 M. G. K. Menon to C. F. Powell, 25 August 1965, TIFR Archives, Mumbai, D‐2004‐00845‐83‐KGF.

n2 M. G. K. Menon to M. H. Parthasarathy, 27 August 1965, TIFR Archives, Mumbai, D‐2004‐00845‐85‐KGF.

n3 M. G. K. Menon to C. F. Powell, 29 August 1965, TIFR Archives, Mumbai, D‐2004‐00845‐86‐KGF.

n4 The strike in August 1965 is reported in “Retrenchment fears to fore,” 1965. The earlier strike began on 17 May 1965 and lasted four days, as reported in “Most Workers Resume Work: Accord at Kolar Gold Mines,” 1965. In response, the public sector mining company agreed to “continue their efforts to procure good quality boiled and other varieties of rice except imported (American) rice.”

n5 A. W. Wolfendale to M. G. K. Menon, 10 November 1965, TIFR Archives, Mumbai, D‐2004‐00845‐103‐KGF. Their Japanese collaborator Saburo Miyake also wrote to Menon in December, reporting that the mines were still shut.

n6 A. W. Wolfendale to M. G. K. Menon, 14 December 1965. TIFR Archives, D‐2004‐00845‐111‐KGF.

n7 M. G. K. Menon to A. W. Wolfendale, 25 December 1965, TIFR Archives, D‐2004‐00845‐112‐KGF. We learn from his letter to Miyake, dated the same day, that the “neutrino level” at KGF was reopened after the fire. Menon suggested that they nevertheless “should use shallower depths since these problems occur at deep levels.” M. G. K. Menon to S. Miyake, 25 December 1965, TIFR Archives, D‐2004‐00845‐113‐KGF. The experiments resumed in March 1966.

n8 Kohler 2002.

n9 A field site has specificities that allow for studies that cannot be conducted elsewhere. The lab–field framework also has a middle ground, the observatory. It is like a field site in that location influences what can be observed. But it is also like a lab in that results are supposed to be replicable, in contrast to a field site where “local variation itself is typically the topic of study.” D. Lane 2008, on 129.

n10 Minor 2020.

n11 Galison and Thompson 1999; Gieryn 1999.

n12 Minor 2020, on 2.

n13 Ibid.

n14 Le Gars and Aubin 2009, on 512.

n15 Ibid.

n16 Brinitzer and Benson 2022; Heggie 2016.

n17 Brinitzer and Benson 2022; Brinitzer 2022.

n18 Heggie 2016, on 825.

n19 Pang 2002.

n20 Brinitzer and Benson 2022, on 110.

n21 Raby 2017.

n22 Raby 2019.

n23 Stark 2022.

n24 Minor 2020.

n25 Kragh 2002, on 193.

n26 Krishnaswamy et al. 1990, on 7.

n27 But by then the mines had been dying, with production decreasing markedly since the 1940s. See Table 4 in Simmons 1985, on 38. The situation was made worse by the Indian government's gold pricing policy, and a corrupt and self‐serving management. The mines were operated by the Mysore state beginning in 1956, and then by the Government of India beginning in 1962 when the public sector Bharat Gold Mines Limited took over, as Kumar et al. 1998 discuss.

n28 Krishnaswamy et al. 1990, on 8.

n29 Miyake et al. 1964; Narasimham 2004, on 12. Physicists from TIFR also studied extended air showers of cosmic rays at KGF above ground, which I do not address in this paper.

n30 This is the process of beta decay. See Riesselmann 2007.

n31 They detected the electron antineutrino created by beta decay.

n32 These are neutrinos of the muon flavor.

n33 Miyake et al. 1964.

n34 Abraham 2000; Abraham 2017.

n35 H. J. Bhabha to F. Reines, 29 July 1963, Frederick Reines Papers (MS.F.007), University of California, Irvine, Special Collections and Archives, Box 60 (Correspondence 1962–1978), Subseries 10.1.

n36 The KGF group used plastic scintillators and neon flash tubes, while the ERPM group used liquid scintillation counters and photomultiplier tubes.

n37 F. Reines, Response to Open Letter, 31 January 1968, Frederick Reines Papers (MS.F.007), University of California, Irvine, Special Collections and Archives, Box 60 (Correspondence 1962–1978), Subseries 10.1.

n38 F. Reines to Paul W. McDaniel, 15 March 1963, Frederick Reines Papers (MS.F.007), University of California, Irvine, Special Collections and Archives, Box 60 (Correspondence 1962–1978), Subseries 10.1.

n39 For example, in 1941, close to 70 percent of the mining workforce were Dalits. Simmons 1990, on 82. Caste can be thought of as a culture‐specific category through which dominance is “recognized, expressed and reproduced but also challenged and resisted.” Aloysius 2010b, on 34. It encodes differential natures that have, for centuries, been considered hereditary in South Asia, and these natures have different social statuses and thus rights and duties. This is the *varna* system, according to which there are four groups, with Brahmins at the top and Shudras at the bottom of the hierarchy. “Untouchable” castes, today called Dalits, are outside this system altogether. These groups came to be associated with different kinds of professions, based on their position in the hierarchy. Thus, as Ambedkar [1936] 1979 notes, caste is not just a division of labor, but also a division of laborers. Two other important features of caste are that it is a graded hierarchy and that caste groups are endogamous.

n40 I use the term “lowered caste” rather than “lower caste” for several reasons. As Mandal 2023 writes, the term “‘lower caste’ […] puts the gaze on the oppressed, implying that their caste status is responsible for their oppression,” whereas the term “‘lowered caste’ puts the blame on the perpetrators and challenges the system's enduring and entrenched nature.” Other reasons are that the latter term “recognises systemic injustice while situating it in history,” “reframes the discussion away from the inherent inferiority of certain castes and toward systemic injustices,” “promotes sensitivity and awareness about caste‐based issues,” and that by “placing the blame on the system, it points to a need for transformation.”

n41 Viswanath 2014.

n42 Nair 1998. However, they faced political neglect because they were linguistic minorities, Tamil speakers in a state where Kannada speakers were the majority.

n43 Thass, as Aloysius 2010a shows, anticipated B. R. Ambedkar and “Periyar” E. V. Ramasamy in his anti‐caste work. Thass had also adopted Buddhism, like Ambedkar later would, and KGF was an important center for the revival of Tamil Buddhism. See Ayyathurai 2023.

n44 Large fans that circulated cool air were installed only in the late 1930s, making the temperature underground bearable.

n45 Simmons 1990, on 94. Around 3,400 miners died in the period 1891–1946. These numbers exclude those who died of occupational diseases such as silicosis. There were fewer accidents and deaths in South African mines: the “death rate” per £100,000 worth of gold produced in the period 1924–1928, on an average, was 3.36 at KGF as opposed to 1.25 in South African mines, as discussed in Sreenivasan 1980, on 67.

n46 Although it had been the de facto standard since 1717. See Schoenberger 2011, on 16.

n47 Ibid. This meant that currencies had a fixed exchange rate.

n48 By the late 1920s, “nearly 70 percent of all gold reserves were held in Britain, France and the U.S.—principally the U.S. which by then held 45 percent of global monetary gold.” Ibid., on 17.

n49 The British empire accounted for 63 percent of global gold production in 1913, with the U.S. at 19 percent. The intense mining meant that “[a]s much gold was produced in the world in the twenty‐odd years from 1894 to 1917 as in the four hundred years between 1492 and 1894.” Spurr 1925, on 478.

n50 The London market price of gold per fine ounce was £4.24 in 1918, virtually unchanged since the early eighteenth century. It rose to £5.90 in 1932 and was close to £9 until 1968. Lawrence H. Officer and Samuel H. Williamson, “The Price of Gold, 1257–Present,” MeasuringWorth, http://www.measuringworth.com/gold/ (accessed 1 March 2024).

n51 Crabbe 1989.

n52 de Witte and Oosterbaan 2013, on 8.

n53 KGF produced, at its peak, 170.8 metric tons of gold in the period 1901–10. See Table 3.1 in Kumar 2009, on 35.

n54 For example, in the period 1901–1910, around 6 million metric tons of ore yielded 170.8 metric tons of gold. This was high‐grade ore, with 28.05 grams of gold extracted from each ton of ore. By the next decade, more ore was mined (nearly 7 million metric tons) but yielded less gold (125.5 metric tons) at a lower grade (18.19 grams/ton). See Table 3.1 in Kumar 2009, on 35.

n55 Van Helten 1980, on 232.

n56 For example, in 1925 and 1938, wages accounted for over 55 percent of the total cost of running the mines. See Table 2 in Simmons 1990, on 103. This remained so until the 1980s, as discussed in Kumar 2009, on 163. The wage bill was nearly 75 percent of the working costs in 1961–62, as shown in *Report* 1967, on 35.

n57
*Report* 1950.

n58 Ibid., on 45.

n59 Ibid., on 46.

n60 Ibid.

n61 Ibid., on 47.

n62 The price of gold per fine ounce increased from £4.25 in 1930 to £5.90 in 1932, further increasing to £8.40 in 1940. It then remained more or less constant until the late 1960s. This is the London market price of gold. See Lawrence H. Officer and Samuel H. Williamson, “The Price of Gold, 1257–Present,” MeasuringWorth, http://www.measuringworth.com/gold/ (accessed 1 March 2024).

n63 Simmons 1990, on 112–113.

n64 Nair 1998.

n65 Table 8 in Simmons 1990, on 112–113.

n66 Tables 4.5 and 4.6 in Kumar 2009, on 80–81.

n67 Europeans, mostly British but also Italians, constituted less than one percent of the workforce, as discussed in Simmons 1990, on 68. There were also “Anglo Indians,” who were of mixed heritage.

n68 Simmons 1990, on 92.

n69 Simmons 1985, on 41.

n70 Kumar et al. 1998, on 1468.

n71 Ibid., on 1469.

n72 Ramu 1979, on 1023.

n73 Chandrashekar 2015.

n74 For instance, in 1929, 56 percent of the underground workers and 25 percent of surface workers, making up 44 percent of the total workforce, were on contract, as discussed in Simmons 1990, on 88.

n75 Nair 1998.

n76 Ibid., on 61.

n77 Ibid.

n78 The experiments at ERPM were discontinued by the early 1970s, the mines having outlived their usefulness for the kinds of experiments now required, and Reines moved on to using mines of lesser depths in the U.S. ERPM, where mining began in the 1890s, shut down in 2008, as mentioned in Decena 2019.

n79 Pinch 1986.

n80 Ibid., on 102.

n81 Ibid., on 103.

n82 See Barbash and Elkind 1980.

n83 Mansell, Roger, “Nagoya POW Camp #1‐B, Kamioka,” Center for Research: Allied POWs Under the Japanese, http://www.mansell.com/pow_resources/camplists/Nagoya/kamioka_1/kamioka.htm (accessed 1 March 2024).

n84 This is the Deep Underground Neutrino Experiment at the Sanford Underground Research Facility.
